# Shelf-Life Evaluation of Ingredient Combinations and Technologies for Use in Pet Food Formulations

**DOI:** 10.3390/ani12020152

**Published:** 2022-01-08

**Authors:** Madison P. Wagoner, Marc R. Presume, Moses E. Chilenje, Gerardo A. Abascal-Ponciano, Jorge L. Sandoval, Hunter R. Smith, Tristan M. Reyes, Barney S. Wilborn, Justin A. Dunavant, Robert P. Mason, Eric K. Altom, Charles W. Starkey, Jason T. Sawyer

**Affiliations:** 1 Department of Animal Sciences, Auburn University, Auburn, AL 36849, USA; mpw0035@auburn.edu (M.P.W.); hzs0101@auburn.edu (H.R.S.); tzr0039@auburn.edu (T.M.R.); wilbobs@auburn.edu (B.S.W.); 2 Department of Poultry Science, Auburn University, Auburn, AL 36849, USA; mrp0067@auburn.edu (M.R.P.); oamoseo@gmail.com (M.E.C.); gaa0029@auburn.edu (G.A.A.-P.); jls0152@auburn.edu (J.L.S.); jad0075@auburn.edu (J.A.D.); cws0031@auburn.edu (C.W.S.); 3 Balchem Animal Nutrition and Health, Balchem Corp., New Hampton, NY 10958, USA; RMason@balchem.com (R.P.M.); EAltom@balchem.com (E.K.A.)

**Keywords:** algin, ground frame, hydrocolloid, pet food, shelf-life, wooden breast

## Abstract

**Simple Summary:**

Creation of new meat-based pet food and pet treats continues to grow at a steady annual rate within the pet food industry. Poultry co-products are often overlooked due to their poor quality and low customer acceptance. However, poultry co-products pose great potential and added value to the pet food industry. Two of the most common poultry co-products (wooden breast and carcass frames) often directed towards pet food were used in a fresh pet food formulation. Due to variations in meat quality because of the wooden breast and carcass frames, a hydrocolloid was utilized to improve fresh pet food characteristics. A hydrocolloid is a type of protein that when added to meat products aids with binding and stabilization of the pet food. For the current study, the combination of sodium alginate and encapsulated calcium lactate pentahydrate (ALGIN) was used. Due to the perceived poor quality of wooden breast and carcass frames, it is plausible that the addition of hydrocolloids can combat the undesirable characteristics. Results from the current study suggest that the impact of ALGIN in poultry co-product pet food combinations does not severely alter shelf-life characteristics of a fresh pet food. However, the inclusion of varying amounts of wooden breast and ground carcass frame can impart a greater impact on shelf-life characteristics in fresh pet food by altering surface color and lipid oxidation.

**Abstract:**

Poultry co-product chicken frames (CF) and wooden breast (WB) along with ingredient technology use may bring enhanced value to the pet food industry. Therefore, the current study focused on evaluating CF and WB combinations along with sodium alginate and encapsulated calcium lactate pentahydrate (ALGIN) inclusion within a fresh pet food formulation under simulated shelf-life conditions. Fresh chicken frames (CF) and boneless-skinless wooden breast (WB) were ground and allocated randomly to one of ten treatment combinations with either 0.5 or 1.0% added ALGIN. Ground treatments were placed into a form and fill vacuum package and stored using a reach-in refrigerated case for 21 days. Packages were evaluated for instrumental surface color, lipid oxidation, water activity, and pH on days 1, 3, 7, 14 and 21 of the display. Packages of pet food were lighter, less red, and more yellow (*p* < 0.05) with increasing percentages of CF regardless of ALGIN inclusion, whereas pH was greater (*p* < 0.05) and lipid oxidation was less (*p* < 0.05) with increasing percentage of WB. Water activity increased (*p* < 0.05) when WB and ALGIN inclusion increased. The current results suggest that the use of ALGIN in a poultry co-product pet food formulation can improve shelf-life characteristics such as surface color and lipid oxidation in fresh pet food.

## 1. Introduction

Creative development and availability of meat-based food and treats for pets in the retail space continue to increase at a rapid pace. According to American Pet Products Association, sales within the U.S. Market in 2020 for pet food and treats alone was $42.0 billion and the estimated sales for 2021 is to be $44.1 billion [1]. Due to the variety of pet food options, pet food customers are provided with an almost limitless number of options based on packaging, price, size of the product, ingredient preference, nutritional benefits, and pet breed options. A focus of the pet food industry has traditionally relied on animal by-products from the rendering industry. All animal material that is considered inedible for human consumption is further processed for animal agriculture purposes such as fertilizer, animal feed and pet food treats and/or food. Type of animal by-products that are rendered include offal, fat, blood, bones, meat trimmings and viscera. Within the poultry meat industry, inedible by-products account for approximately 28% of the live weight of a broiler chicken [2]. Poultry co-products are often undervalued throughout the meat industry due to their poor quality and low customer acceptance. However, it is plausible that these co-products could be a potential key component in adding value to the pet food industry.

The poultry industry has struggled with a muscle myopathy identified as wooden breast (WB) resulting in tough muscle texture. This muscle abnormality has and continues to have a negative impact economically on the broiler industry [3]. WB is characterized as a myopathy that presents an overtly harder chicken breast, with sections being swollen and pale resulting in decreased meat quality, yield, and consumer/customer acceptance [4]. Initially, visual characterization of WB was identified by the white striations throughout the breast meat [5] because of excessive accumulation of lipids and connective tissue [6]. Severely affected chicken breasts caused by muscle fiber degeneration have been downgraded by the processor and directed to be further processed within the meat and poultry industry that may include deli meats, sausages, emulsified products, and even pet food. Pet food buyers are influenced by similar organoleptic attributes when buying food for their pets such as aroma, texture, and color. In addition, uniformity and branding are important drivers that may suggest higher quality. Studies have reported that pet food customers are highly influenced by the appearance of the pet food or treat and a darker brown color seems to influence pet owner purchasing intent [7]. Another poultry by-product gaining consideration for use in possible pet food formulations is chicken frames (CF) because of it is nutritional properties [8]. However, when nutritionally evaluating chicken meat and bone meals, these co-products consist of a greater protein level (50 percent) and contain more saturated fatty acids when compared to other rendered meat [9].

Due to the low quality of WB and CF, it is plausible that the addition of hydrocolloids to these meat co-products may aid in creating more desirable characteristics making the co-products suitable for fresh pet food and pet treat applications. Hydrocolloids are used to define a range of proteins and polysaccharides that contain an affinity for water. Hydrocolloids are used throughout the meat and food industry to aid in a variety of formulation functions that include gelling, binding, coating, thickening, stabilization of pH, enhanced heat resistance, salt tolerance, as well as reduce undesirable effects caused when reducing fat and salt content [10,11,12]. Interestingly, gelling of food products via hydrocolloids allows for the product to become more stable [13]. Out of the wide range of hydrocolloids within the meat and food industry today, the combination of sodium alginate and encapsulated calcium lactate pentahydrate (ALGIN) was selected for the current study. Sodium alginate has been commonly used within the food industry in the development of functional food products for human and companion animal consumption. Sodium alginate is an irreversible hydrocolloid that is derived from brown algae, a polysaccharide that is composed of 1–4, β-D mannuronic acid and α-L guluronic acid sugar residues [14,15]. An advantage of using the algin/calcium gel combination, has reported improvements in binding cooked or raw meat products and can prevent the potential negative effects of added salt on product quality [16,17,18]. To our knowledge, there is very little literature about the effects of ALGIN when included in poultry co-product formulations such as CF and WB with the intended purpose of creating pet food products. Therefore, the aim of this study was to evaluate the inclusion rate of ALGIN on meat co-product formulations of CF and WB and subsequent impact on fresh pet food shelf-life characteristics.

## 2. Materials and Methods

### 2.1. Raw Materials

Boneless-skinless wooden breast (WB) meat and de-boned chicken frames (CF) were purchased from a commercial poultry processing facility in North Alabama. Classification of WB was conducted by trained plant personnel at the time of grading using the scoring methods described in [19]: (0 = normal; 1 = moderate, 2 = severe; and 3 = extreme). Fresh, raw materials were transported under refrigerated conditions (2 °C) to the Lambert-Powell Meats Laboratory at Auburn University and stored in the absence of light at 2 °C for 36 h. Fresh, raw poultry materials WB and CF were ground twice using a commercial meat grinder (Hollymatic 3000, Thompson Meat Machinery, Queensland, Australia). To aid in reducing the surface area of the raw materials, CF was ground once through a 9.52 mm grinder plate then a second time through a 4.76 mm grinder plate (SPECO 400, Schiller Park, IL, USA). Fresh boneless-skinless WB meat was ground twice through a 4.76 mm grinder plate (SPECO 400, Schiller Park, IL, USA). After grinding, ground WB and CF were weighed and randomly allocated to 10 treatment batches (N = 22.67 kg/treatment) with two replications (*n* = 11.34 kg/replication). Treatment combinations (Table 1) of WB and CF (COMB) were mixed in a commercial mixer (AFMG 48, Biro, Marblehead, OH, USA) for 5 min while slowly incorporating sodium alginate and calcium lactate (ALGIN). After mixing, each treatment was portioned into 454 ± 0.5 g bricks using a vacuum stuffer (Model VF608plus, Handtmann, Biberach, Germany).

### 2.2. Treatment Packaging

After portioning, fresh pet food bricks were packaged using a Reiser form and fill vacuum packaging machine (Optimus OL0924, Variovac, Zarrentin, Germany). Each package was placed into a commercial film (WINPAK, Winnipeg, MB, Canada) that was comprised of a forming film with a standard barrier consisting of 175 μm Nylon, EVOH and enhanced polyethylene coextrusion and a non-forming film layer was comprised of 60 μm Nylon, EVOH and polyethylene coextrusion. The oxygen transmission rates (OTR) for the forming and non-forming films were 0.4 cc/sq. m/24 h and 1.2 cc/sq. m/24 h, respectively.

### 2.3. Simulated Retail Display Conditions

Packages of fresh pet food were stored in a pull-door, self-service refrigerated 3 tiered LED lighted display case (Model 178GDC49HCB, Avantco Refrigeration, Lancaster, PA, USA) for up to 21 days. Storage temperatures during the simulated display were monitored using a data-recording device (TD2F, Thermoworks, American Fork, UT, USA) with probes placed within the center of each shelf. Refrigerated case temperatures averaged 2.1 ± 1.25 °C. The continuous LED (308 lux) lighting of each shelf was measured in the display cases with a hand-held light meter (Model ILT10C, International Light Technologies, Peabody, MA, USA). Packages of pet food were distributed evenly across the shelving and rotated daily from top to bottom and front to back within the display cooler to reduce temperature variation and simulate consumer package shifting at the retail counter.

### 2.4. Instrumental Color Measurement

Instrumental color readings were collected by scanning each sample through the packaging [20], at three separate locations on each package using a HunterLab MiniScan XE Plus Colorimeter (Model 45/0-L, Hunter Associates Laboratory Inc., Reston, VA, USA). Color readings (L*, a* and b*) were recorded using Illuminant D65, a 10° observer with a 25 mm diameter aperture using the Commission Internationale de l’ Eclairage (CIE L*a*b*) color scale [21]. Prior to capturing objective surface color readings for fresh color, the colorimeter was calibrated on each sampling day 1, 3, 7, 14, and 21 using the standard black and white tiles (L*, 0 = black, 100 = white; a*, −60 = green, +60 = red; b*, −60 = blue, and +60 = yellow).

### 2.5. Thiobarbituric Acid Reactive Substances (TBARS)

On days 1, 3, 7, and 14 of the simulated display period, packages of fresh pet were removed from the display cases and frozen at −80 °C until TBARS analysis could be completed. Prior to analysis, samples were placed into a refrigerated cooler 4 °C and thawed for 12 h. After thawing, pet food was removed from the packaging material and prepared for 2-thiobarbituric acid reactive substance using a modified version [21]. In duplicate, approximately 5 g of each package was homogenized with 8 mL of cold (1 °C) of 50 mM phosphate buffer (pH of 7.0 at 4 °C) that contained 0.1% ethylenediaminetetraacetic acid, 0.1% n-propyl gallate, and 2 mL trichloroacetic acid (Sigma-Aldrich, Saint Louis, MO, USA). After homogenizing, samples were filtered through Whatmann No.4 filter paper and duplicate 2 mL aliquots of the clear supernatant were transferred into 10 mL borosilicate tubes, mixed with 2 mL of 0.02 M 2-thiobarbituric acid reagent (BeanTown Chemical, Hudson, NH, USA), and boiled at 100 °C for 20 min. Immediately after boiling, tubes were placed into an ice bath for 15 min. Finally, absorbance was measured at 533 nm with a spectrophotometer (Turner Model—SM110245, Barnstead International, Dubugue, IA, USA) and then multiplied using a factor of 12.21 to derive the TBARS value (mg of malonaldehyde/kg of fresh meat) [22]. The value of 12.21 was obtained previously from a standard curve using a known malonaldehyde solution measured across multiple absorbencies.

### 2.6. Fresh pH and Water Activity (a_w_)

Packages of fresh pet food from each treatment were chosen randomly from the display cabinets on days 1, 3, 7, 14, and 21 to be analyzed for pH and water activity (a_w_). Prior to collecting pH readings, the pH meter was calibrated using 2-point standard buffers (pH 4.0 and 7.0). Duplicate packages from each treatment were opened and using a pH electrode attached to a pH meter (Model HI199163, Hanna Instruments, Woonsocket, RI, USA) was inserted into the ground pet food. Measurements for pH were collected in triplicate from each packaged and averaged. For water activity samples, 4 g of ground pet food was removed from each package in duplicate inserted into the plastic sample container and analyzed using a benchtop water activity meter AUQALAB 4TE (Dew Point Model, METER Group, Inc., Pullman, WA, USA) which uses the dew point principal method [23].

### 2.7. Statistical Analysis

An analysis of variance was computed using a generalized linear mixed model (GLIMMIX) procedure with statistical analysis software (SAS Institute, Inc. Cary, NC, USA) version 9.4. Fixed effects for ground chicken frames (CF) and wooden breast (WB) and ALGIN inclusion percentage along with their interaction were evaluated. Least-squares means were computed for all variables, and when significant (*p* ≤ 0.05) F-values were observed, least-squares means were separated using pair-wise *t*-tests (PDIFF option).

## 3. Results and Discussion

### 3.1. Instrumental Fresh Color

The combination (COMB) of WB and CF and ALGIN across DAY of simulated display presented an interaction (*p* < 0.05) for instrumental fresh surface color lightness (Table 2). Surface color of pet food packages was lighter (*p* < 0.05) with greater percentages of CF regardless of ALGIN or day of display. However, as the duration of display increased, surface color lightness became darkest (*p* < 0.05) with increasing percentages of WB (Table 2). In addition, an interaction of COMB × ALGIN × DAY occurred for instrumental surface color redness (Table 3). Packages of pet food formulations with greater percentages of WB were redder initially (*p* < 0.05), whereas packages of CF remained lighter throughout the entire display period regardless of ALGIN inclusion (Table 3). Lastly, an interactive influence of COMB × ALGIN × DAY for surface color yellowness occurred (Table 4). Packages of pet food formulations were more yellow (*p* < 0.05) throughout the entire display period when the percentage of CF was greatest and ALGIN was only 0.5% within the formulation. Moreover, as the concentration of WB increased, surface b* became greener (*p* < 0.05). Fresh surface color of pet food remains an enigma within the retail market because the intended user of the food is not visually appraising the product in the same manner fresh edible meat products are assessed. However, it is plausible that purchasers of fresh pet food (pet owners) will continue to use surface color as an indicator of wholesome and freshness of pet food constructed with fresh meat ingredients that can deteriorate during a storage period. In a similar study [24] on surface color differences between cooked and raw WB samples with non-affected boneless, skinless, breast fillets, it was reported that the chicken breasts with severe WB can have greater redness (a*) values, which appears consistent with our findings in the current study. In additional studies, it has been determined that boneless, skinless WB fillets often have more connective tissue and a greater percentage of white striations throughout the filet often causing an altered surface color of the meat [5,24,25,26]. It was expected that the formulations with higher percentages of WB would result in greater lightness (L*) values due to less muscle myoglobin influence and greater hemorrhagic lesions throughout the affected WB meat. However, the current results indicate lightness (L*) values for treatment combinations containing more WB in the formulations darker, redder and greener. A similar reported that WB fillets had significantly greater (*p* < 0.05) lightness values (L*) and yellowness values (b*), which are inconsistent with our findings [25]. The inconsistency in surface color results may be attributed to the addition of CF, severity of WB within the COMB, but does not suggest that ALGIN imparted surface color changes throughout the simulated display period.

### 3.2. Thiobarbituric Acid Reactive Substances (TBARS)

An interaction (*p* < 0.05) of COMB × ALGIN × DAY for TBARS values occurred during the simulated retail display period (Table 5). As a result of fresh pet food quality declining throughout the display period, TBARS values were only measured through 14 days of the simulated display. TBARS values were greatest (*p* < 0.05) on day 14 when ALGIN and WB combinations were 1.0 and 100%, respectively. With limited results available from previous studies, the values from the current study provide a foundation to lipid oxidation changes that may occur during storage of fresh pet food. Previous lipid oxidation findings for WB have suggested that oxidation can be variable [27], whereas frozen storage of cooked chicken sausage formulated with WB can range from 0.14 to 2.00 mg malonaldehyde/kg [28]. Countless studies in chicken [29,30] and beef [31,32] suggest TBARS values may exceed 3 mg of malonaldehyde/kg in fresh or cooked meat samples. Current values for TBARS values align with previous studies and provide a baseline for future studies. Consumer perception of meat products for wholesomeness and freshness at the time of use may be partly influenced by lipid oxidation [33]. However, it is necessary that additional research on fresh pet food shelf-life using various ingredient technologies that may or may not improve lipid oxidation be investigated.

### 3.3. Fresh Pet Food pH Values

There was an interactive (*p* < 0.05) effect of COMB × ALGIN × DAY on pH values of fresh pet food during a simulated retail display (Table 6). Pet food containing more than 75% WB in the formulation resulted in greater (*p* < 0.05) pH values throughout the 21 day simulated retail period. It has been reported that WB tends to have greater pH values because of muscle degeneration and implications on glycogen content minimizing lactic acid formation in postmortem muscle [34,35,36]. It has been noted that CF pH ranges tend to fall within 6.5 to 6.9 [37]. It is plausible that the variation noted in surface color of the current study are attributed to the influences of fresh pH values.

### 3.4. Water Activity (a_w_)

There was no interactive (*p* > 0.05) impact of COMB × ALGIN × DAY on water activity during the simulated retail display. However, an interactive influence of COMB × ALGIN (Table 7) on water activity occurred. Water activity increased with increasing usage of ALGIN and percentage of WB in the formulation. As expected, ALGIN improved water holding capacity as WB inclusion increased. It is known [38] that water holding capacity is less in WB leading to further implications on water activity. In addition, there was a COMB × DAY interaction (*p* < 0.05) on water activity (Table 8). Ground fresh pet food with a greater percentage of WB resulted in greater water activity throughout the simulated retail display. Lastly, the interactive (*p* < 0.05) effect of ALGIN × DAY on water activity provides further support that the use of hydrocolloids in a meat system can aid water retention (Table 9). At the conclusion (day 21) of simulated retail display, water activity was greater (*p* < 0.05) in fresh pet food containing 1.0% ALGIN. Previous results support the use of hydrocolloids in a meat system for improving water retention [39].

## 4. Conclusions

Little to no information regarding fresh pet food shelf-life studies currently exists within the research arena. Therefore, presentation of current findings provides a brief snapshot into the use of current ingredient technologies from the meat and food industries that may be considered as viable tools for formulating fresh pet food. The current results suggest that the inclusion of ALGIN on poultry co-product pet food combinations involving CF and/or WB can improve fresh surface color characteristics. However, the combination of CF or WB used can alter surface color lightness and redness regardless of ALGIN. Regardless, the current results suggest that ALGIN with either CF or WB can be utilized in the formulation of a fresh pet food. Additional research focused on the optimal shelf-life storage period of fresh pet food is needed.

## Figures and Tables

**Table 1:** Pet food formulations with poultry co-product and ALGIN ^1^ inclusion percentages.

Treatments										
Ingredients	A	B	C	D	E	F	G	H	I	J
Wooden Breast, %	0.00	0.00	25	25	50	50	75	75	100	100
Chicken Frame, %	100	100	75	75	50	50	25	25	0.00	0.00
Total, %	100	100	100	100	100	100	100	100	100	100
ALGIN ^1^										
Sodium Alginate, %	1.00	0.50	1.00	0.50	1.00	0.50	1.00	0.50	1.00	0.50
Calcium Lactate, %	0.85	0.425	0.85	0.425	0.85	0.425	0.85	0.425	0.85	0.425

^1^ ALGIN is an inclusion percentage (%) of two functional ingredients, sodium alginate and calcium lactate.

**Table 2:** Interactive effect of COMB × ALGIN × DAY on instrumental fresh color lightness (L*) ^1^ of fresh pet food formulations during a simulated retail display.

COMB ^2^											
	100 CF:00 WB		75 CF:25 WB		50 CF:50 WB		25 CF:75 WB		00 CF:100 WB		
ALGIN ^3^											
	0.5	1.0	0.5	1.0	0.5	1.0	0.5	1.0	0.5	1.0	SEM
Day 1 ^4^	62.88 ^cd^	62.68 ^cd^	57.54 ^hij^	57.04 ^ijk^	56.13 ^klm^	54.91 ^nopqr^	53.89 ^stuv^	52.34 ^xyz^	52.63 ^wxy^	51.70 ^yza*^	0.4993
Day 3	62.63 ^cd^	63.27 ^c^	58.00 ^ghi^	57.29 ^ij^	56.61 ^jkl^	55.23 ^mnop^	54.98 ^nopq^	53.72 ^tuv^	54.45 ^opqrstu^	54.25 ^pqrstu^	0.4993
Day 7	59.17 ^f^	60.22 ^e^	53.78 ^tuv^	54.64 ^opqrst^	52.06 ^xyz^	51.76 ^yza*^	51.35 ^za*^	50.81 ^a*b*^	50.83 ^a*b*^	50.06 ^b*^	0.4993
Day 14	64.80 ^b^	66.03 ^a^	59.15 ^f^	58.83 ^fg^	58.47 ^fgh^	57.02 ^ijk^	54.81 ^nopqrs^	55.42 ^mno^	53.70 ^tuv^	53.98 ^rst^	0.4993
Day 21	62.09 ^d^	64.71 ^b^	57.01 ^jk^	57.14 ^ij^	55.64 ^lmn^	54.58 ^opqrst^	53.48 ^uvw^	54.04 ^qrstu^	54.35 ^pqrstu^	52.96 ^vwx^	0.4993

^1^ Lightness (L*) values are a measure of darkness to lightness (larger value indicates a lighter color). ^2^ COMB is the raw material formulation of ground chicken frame (CF) and/or boneless-skinless wooden breast (WB). ^3^ ALGIN is the inclusion percentage (%) of two functional ingredients, sodium alginate and calcium lactate. ^4^ Simulated storage conditions consisted of reach-in refrigerated cabinet maintained at 4 °C. ^a–b*^ All mean values lacking common superscripts differ (*p* < 0.05).

**Table 3:** Interactive effect of COMB × ALGIN × DAY on instrumental fresh color redness (a*) ^1^ of fresh ^1^ Lightness (L*) values are a measure of darkness to lightness (larger value indicates a lighter color).

COMB ^2^											
	100 CF:00 WB		75 CF:25 WB		50 CF:50 WB		25 CF:75 WB		00 CF:100 WB		
ALGIN ^3^											
	0.50	1.00	0.50	1.00	0.50	1.00	0.50	1.00	0.50	1.00	SEM
Day 1 ^4^	6.42 ^a*^	6.14 ^a*^	9.86 ^w^	9.91 ^w^	11.18 ^nopqrs^	11.43 ^jklmnop^	11.98 ^defghi^	12.32 ^bcd^	12.61 ^ab^	12.87 ^a^	0.2603
Day 3	6.58 ^za*^	6.30 ^a*^	10.03 ^w^	10.17 ^vw^	11.28 ^lmnopqr^	11.81 ^efghijk^	12.17 ^bcdefg^	12.07 ^cdefghi^	12.12 ^bcdefg^	12.21 ^bcdef^	0.2603
Day 7	7.11 ^y^	7.07 ^yx^	10.65 ^tuv^	10.27 ^uvw^	11.79 ^fghijkl^	11.60 ^hijlkmno^	12.01 ^cdefgh^	12.25 ^bcdefg^	11.69 ^ghijklmn^	12.46 ^abcd^	0.2603
Day 14	7.80 ^x^	7.09 ^yz^	10.77 ^rstu^	11.27 ^mnopqr^	11.76 ^fghijklm^	11.87 ^efghij^	11.94 ^defghij^	11.88 ^efghij^	12.53 ^abc^	11.56 ^ijklmnop^	0.2603
Day 21	6.51 ^a*^	6.27 ^a*^	9.86 ^w^	9.95 ^w^	10.85 ^qrst^	11.30 ^klmnopq^	11.19 ^nopqrs^	11.09 ^opqrst^	11.07 ^pqrst^	10.75 ^stu^	0.2603

^1^ Lightness (L*) values are a measure of darkness to lightness (larger value indicates a lighter color). ^2^ COMB is the raw material formulation of ground chicken frame (CF) and/or boneless-skinless wooden breast (WB). ^3^ ALGIN is the inclusion percentage (%) of two functional ingredients, sodium alginate and calcium lactate. ^4^ Simulated storage conditions consisted of reach-in refrigerated cabinet maintained at 4 °C. ^a–b*^ All mean values lacking common superscripts differ (*p* < 0.05).

**Table 4:** Interactive effect of COMB × ALGIN × DAY on instrumental fresh color yellowness (b*) ^1^ of pet food formulations during a simulated retail display.

COMB ^2^											
	100 CF:00 WB		75 CF:25 WB		50 CF:50 WB		25 CF:75 WB		00 CF:100 WB		
ALGIN ^3^											
	0.5	1.0	0.5	1.0	0.5	1.0	0.5	1.0	0.5	1.0	SEM
Day 1 ^4^	21.73 ^cde^	20.78 ^f^	18.37 ^hi^	18.21 ^hi^	16.41 ^pqrs^	16.92 ^nop^	15.84 ^tuv^	15.15 ^xy^	15.73 ^uvw^	15.24 ^wxy^	0.2853
Day 3	22.09 ^bcd^	21.57 ^de^	18.48 ^h^	18.52 ^h^	17.02 ^mno^	17.27 ^lmn^	16.60 ^opq^	15.42 ^vwx^	15.71 ^uvw^	15.46 ^vwx^	0.2853
Day 7	21.47 ^e^	21.35 ^e^	18.04 ^hij^	17.49 ^jklm^	16.33 ^qrst^	16.38 ^pqrst^	16.15 ^rstu^	15.02 ^xy^	14.84 ^xy^	15.13 ^xy^	0.2853
Day 14	23.70 ^a^	22.57 ^b^	19.57 ^g^	19.61 ^g^	17.59 ^jkl^	17.62 ^jkl^	17.85 ^ijk^	16.41 ^pqrs^	16.91 ^nop^	16.59 ^opqr^	0.2853
Day 21	22.25 ^bc^	21.29 ^ef^	18.56 ^h^	18.46 ^h^	17.35 ^klmn^	17.15 ^lmno^	16.87 ^nopq^	15.89 ^stuv^	15.50 ^vwx^	16.34 ^qrst^	0.2853

^1^ Yellowness (b*) values are a measure of darkness to lightness (larger value indicates a more yellow color). ^2^ COMB is the raw material formulation of ground chicken frame (CF) and/or boneless-skinless wooden breast (WB). ^3^ ALGIN is the inclusion percentage (%) of two functional ingredients, sodium alginate and calcium lactate. ^4^ Simulated storage conditions consisted of reach-in cabinet coolers maintained at 4 °C. ^a–y^ All mean values lacking common superscripts differ (*p* < 0.05).

**Table 5:** Interactive effect of COMB × ALGIN × DAY on TBARS ^1^ value of pet food formulations during a simulated retail display.

COMB ^2^											
	100 CF:00 WB		75 CF:25 WB		50 CF:50 WB		25 CF:75 WB		00 CF:100 WB		
ALGIN ^3^											
	0.5	1.0	0.5	1.0	0.5	1.0	0.5	1.0	0.5	1.0	SEM
Day 1 ^4^	1.87 ^efghij^	1.72 ^hijk^	1.88 ^efghij^	1.85 ^efghhij^	1.92 ^efghi^	1.79 ^fghij^	2.53 ^abcd^	1.71 ^hijk^	1.65 ^hijk^	0.84 ^l^	0.3042
Day 3	2.69 ^abc^	1.77 ^fghij^	1.79 ^fghij^	1.80 ^fghij^	2.73 ^abc^	1.87 ^efghij^	1.83 ^fghij^	1.35 ^ijkl^	1.29 ^jkl^	1.60 ^hijk^	0.3042
Day 7	1.89 ^efghij^	1.71 ^hijk^	2.37 ^bcdef^	2.34 ^bcdefg^	2.95 ^ab^	1.15 ^lk^	2.46 ^abcde^	2.19 ^cdefgh^	0.89 ^l^	1.67 ^hijk^	0.3042
Day 14	2.56 ^abcd^	2.46 ^abcde^	1.97 ^defgh^	1.87 ^efghhij^	1.64 ^hijk^	1.98 ^defgh^	1.74 ^ghijk^	1.82 ^fghij^	0.87 ^l^	3.05 ^a^	0.3042

^1^ 2-Thiobarbituric acid reactive substance (TBARS) are a measure of lipid oxidation, with a larger value indicating a greater amount of oxidation (mg malonaldehyde/kg ^−1^ of fresh meat). ^2^ COMB is the raw material formulation of ground chicken frame (CF) and/or boneless-skinless wooden breast (WB). ^3^ ALGIN is the inclusion percentage (%) of two functional ingredients, sodium alginate and calcium lactate. ^4^ Simulated storage conditions consisted of reach-in cabinet coolers maintained at 4 °C. ^a–l^ Mean values lacking common superscripts differ (*p* < 0.05).

**Table 6:** Interactive effect of COMB × ALGIN × DAY on pH of pet food formulations during a simulated retail display.

COMB ^1^											
	100 CF:00 WB		75 CF:25 WB		50 CF:50 WB		25 CF:75 WB		00 CF:100 WB		
ALGIN ^2^											
	0.5	1.0	0.5	1.0	0.5	1.0	0.5	1.0	0.5	1.0	SEM
Day 1 ^3^	6.13 ^stu^	6.01 ^tuv^	6.48 ^ijklmno^	6.52 ^ijkl^	6.84 ^cde^	6.76 ^defg^	7.06 ^b^	6.92 ^bcd^	7.41 ^a^	7.38 ^a^	0.0824
Day 3	6.10 ^stu^	6.16 ^qrstu^	6.49 ^ijklmn^	6.39 ^klmnop^	6.59 ^ghij^	6.62 ^fghi^	6.84 ^cde^	6.69 ^efgh^	6.82 ^cde^	6.98 ^bc^	0.0824
Day 7	5.81 ^xyz^	5.80 ^xyz^	5.89 ^wxy^	5.88 ^wxyz^	6.12 ^stu^	5.93 ^vwx^	6.25 ^pqrs^	6.36 ^lmnop^	6.51 ^ijklm^	6.39 ^klmnop^	0.0824
Day 14	5.86 ^xyz^	5.75 ^yz^	6.04 ^tuvw^	6.01 ^tuv^	6.15 ^rstu^	6.15 ^rstu^	6.44 ^jklmno^	6.22 ^pqrst^	6.35 ^mnop^	6.39 ^klmnop^	0.0824
Day 21	6.01 ^tuvw^	5.72 ^z^	6.53 ^ijk^	6.32 ^opqr^	6.02 ^uvw^	6.77 ^def^	6.33 ^nopq^	6.35 ^mnop^	6.58 ^hij^	6.34 ^nop^	0.0824

^1^ COMB is the raw material formulation of ground chicken frame (GF) and/or boneless-skinless wooden breast (WB). ^2^ ALGIN is the inclusion percentage (%) of two functional ingredients, sodium alginate and calcium lactate. ^3^ Simulated storage conditions consisted of reach-in cabinet coolers maintained at 4 °C. ^a–z^ All mean values lacking common superscripts differ (*p* < 0.05).

**Table 7:** Interactive effect of COMB × ALGIN on water activity (a_w_) of pet food formulations during a simulated retail display.

			COMB ^1^			
	100 CF:00 WB	75 CF:25 WB	50 CF:50 WB	25 CF:75 WB	00 CF:100 WB	SEM
AGLIN ^2^ 0.5	0.994 ^dc^	0.993 ^e^	0.993 ^de^	0.994 ^dc^	0.995 ^bc^	0.000476
ALGIN 1.0	0.993 ^e^	0.994 ^dc^	0.993 ^de^	0.996 ^a^	0.995 ^ab^	0.000476

^1^ COMB is the raw material formulation of ground chicken frame (CF) and/or boneless-skinless wooden breast (WB). ^2^ ALGIN is the inclusion percentage (%) of two functional ingredients, sodium alginate and calcium lactate. ^a–e^ Mean values lacking common superscripts differ (*p* < 0.05).

**Table 8:** Interactive effect of COMB × DAY on water activity (a_w_) of pet food formulations during a simulated display.

	COMB ^1^					
	100 CF:00 WB	75 CF:25 WB	50 CF:50 WB	25 CF:75 WB	00 CF:100 WB	SEM
Day 1 ^2^	0.992 ^ijk^	0.992 ^jkl^	0.991 ^l^	0.993 ^ghij^	0.993 ^fghij^	0.000753
Day 3	0.996 ^b^	0.996 ^bcd^	0.996 ^bc^	0.997 ^ab^	0.998 ^a^	0.000753
Day 7	0.993 ^ghij^	0.992 ^ijk^	0.992 ^ijk^	0.994 ^fghij^	0.994 ^defg^	0.000753
Day 14	0.991 ^l^	0.991 ^kl^	0.993 ^hijk^	0.994 ^efghi^	0.994 ^efghi^	0.000753
Day 21	0.993 ^fghij^	0.995 ^efgh^	0.995 ^cdef^	0.997 ^ab^	0.995 ^cde^	0.000753

^1^ COMB is the raw material formulation of ground chicken frame (CF) and/or boneless-skinless wooden breast (WB). ^2^ Simulated storage conditions consisted of reach-in cabinet coolers maintained at 4 °C. ^a–l^ Mean values lacking common superscripts differ (*p* < 0.05).

**Table 9:** Interactive effect of ALGIN × DAY on water activity (a_w_) on pet food formulations during a simulated retail display.

	DAY					
	1	3	7	14	21	SEM
ALGIN ^1^ 0.5	0.993 ^ef^	0.997 ^a^	0.993 ^de^	0.992 ^f^	0.995 ^c^	0.000476
ALGIN 1.0	0.993 ^def^	0.997 ^ab^	0.994 ^cd^	0.994 ^cde^	0.996 ^b^	0.000476

^1^ ALGIN is the inclusion percentage (%) of two functional ingredients, sodium alginate and calcium lactate. ^a–f^ Mean values lacking common superscripts differ (*p* < 0.05).

## Data Availability

Not Applicable.
